# Updates on the Status of Carbapenem-Resistant Enterobacterales in Lebanon

**DOI:** 10.1155/2023/8831804

**Published:** 2023-05-29

**Authors:** Mahdi Fadlallah, Ahmad Salman, Elie Salem-Sokhn

**Affiliations:** ^1^Laboratory Medicine, Lebanese University, Faculty of Medical Sciences, Beirut, Lebanon; ^2^Infectious Diseases, Lebanese University, Faculty of Medical Sciences, Beirut, Lebanon; ^3^Department of Medical Laboratory Technology, Faculty of Health Sciences, Beirut Arab University, P.O. Box 11-5020, Beirut, Lebanon

## Abstract

Carbapenem-resistant Enterobacterales (CRE) pathogens have been increasingly isolated and reported in Lebanon. Several studies have been published over the last two decades about the CRE situation in the country. However, compared to the worldwide data, those studies are scarce and mostly restricted to single center studies. In this review, we aim to present a comprehensive and reliable report illustrating the current situation regarding CRE in Lebanon. Variable studies have shown an increasing pattern of carbapenem resistance in Enterobacterales since the first reports of CRE isolates in 2007 and 2008. *Escherichia coli* and *Klebsiella pneumoniae* were the most detected ones. The OXA-48 class D carbapenemases were the most prevalent carbapenemases among CRE isolates. Moreover, the emergence of other carbapenemases like the NDM class B carbapenemase has been noticed. Strict infection control measures in hospitals, including the identification of CRE carriers, are needed in Lebanese hospitals since carriage is a potential risk for the spread of CRE in healthcare settings. The dissemination of CRE in the community is noticed and attributed to multiple causes, such as the refugee crisis, water contamination, and antimicrobial misuse. In conclusion, strict infection control measures in healthcare settings, in addition to accurate antimicrobial stewardship program implementation, are urgently needed.

## 1. Introduction

Emergence of antimicrobial resistance (AMR) is a serious global and public health burden [1]. AMR was recognized as “a global health security threat” for which action is needed across society and government sectors [2]. Increased mortality and morbidity with costlier and prolonged treatments and hospitalization have been associated with antibiotic-resistant bacteria infections. Consequently, the Centers for Disease Control and Prevention (CDC) has estimated direct health costs related to AMR as high as $20 billion in the United States [3]. In 2017, the World Health Organization (WHO) published a list of bacteria with emergent AMR, which was categorized into 3 groups according to the priority of need for new antibiotics: critical, high, and medium priority. Carbapenem-resistant Enterobacterales (CRE) organisms are among the critical priority groups [4]. Carbapenems are considered the last-line treatment option for infections caused by multidrug-resistant bacteria (MDR) [5, 6]. Thus, very limited treatment options for these infections are available to date [7].

In 2015, CDC defined CRE as Enterobacterales that are nonsusceptible (resistant or intermediate) in vitro to any of the carbapenems or those documented to produce a carbapenemase [8, 9]. CRE is further divided into carbapenemase producers-CRE (CP-CRE) and nonproducers [8]. Due to the rapid spread in healthcare systems, CP-CRE are considered an important public health and clinical concern [10] where its spread most commonly occurs in hospitals, nursing homes, and other healthcare setting [11].

An increasing number of carbapenem-resistant *Escherichia coli* (*E. coli*) and *Klebsiella pneumoniae* (*K. pneumoniae*) during 2016–2020 have been reported in Europe [12]. In 2017, an estimate of 13,100 cases of CRE were in hospitalized patients, with 1,100 deaths announced in the United States [11]. However, in Lebanon, as in other countries in the Middle East, data about the epidemiology and distribution of carbapenem resistance are scarce [13] and mostly restricted to single center studies [14]. In light of the serious clinical threats that CRE is causing and the scarcity of reliable data, we intend to review the most important studies concerning carbapenem resistance in Enterobacterales in Lebanon. Our aim is to present the available data regarding the current situation of CRE characteristics and prevalence in Lebanon.

## 2. Types of CRE

**Figure 1:**
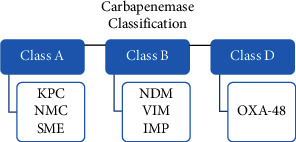
Classification of carbapenemases *β*-lactamases that confer resistance to carbapenems in Enterobacterales (reference 5). KPC: *Klebsiella pneumoniae* carbapenemase; IMP: imipenemase metallo-*β*-lactamase; NDM: New Delhi metallo-*β*-lactamase; NMC: nonmetallocarbapenemase; OXA: oxacillin carbapenemase/oxacillinase; SME: *Serratia marcescens* enzyme; VIM: Verona integron-encoded metallo-*β*-lactamase.

Carbapenemase production has been reported as the major cause of carbapenem resistance in Enterobacterales [15]. A large number of carbapenemase has been reported in Enterobacterales, belonging to three classes of beta-lactamase defined by Ambler classification: Class A, Class B, and Class D (Figure 1). The most common class A carbapenemase is *Klebsiella pneumoniae* carbapenemase (KPC) and has been reported as the most common carbapenemase in the US [5, 16]. Class B carbapenemases are metallo-*β*-lactamases (MBLs) that hydrolyze carbapenem in a zinc dependent manner and includes New Delhi metallo-*β*-lactamases (NDMs), imipenemase metallo-*β*-lactamase (IMP), and Verona integron-encoded metallo-*β*-lactamases (VIMs) [5, 17]. Class D carbapenemases include exclusively OXA-48 enzymes. These enzymes are difficult to detect, characterized by weak hydrolysis of carbapenem and are widespread in the Middle East, North Africa, and Europe [5, 17]. However, carbapenem resistance due to mechanisms other to carbapenemase production has been reported. Overexpression of AmpC enzymes and extended spectrum beta lactamase (ESBL) production associated with porin loss have been also mentioned as a cause of carbapenem resistance in Enterobacterales [17, 18].

## 3. CRE Reports in Lebanon (Table 1)

CRE has become an alarming threat to the Lebanese community. Isolates cultured from different tissue sites and from different hospitals all over Lebanon are increasingly reported. The first comprehensive report to study bacterial antibiotic susceptibility was done at the American University of Beirut Medical Center (AUBMC). A total of 3773 Gram-negative bacteria were examined between March 1992 and 30 June 1993. All Enterobacterales, including *Citrobacter* spp., *Enterobacter* spp., *E. coli*, *Proteus* spp., and *Serratia* spp., were found to be 100% susceptible to imipenem [19].

Data from that time were scarce and it was not until the years 2007-2008 that more reports on carbapenem-resistant Enterobacterales started to be published. Of note, the isolation of the first MBL-producing* K. pneumoniae* in Lebanon was reported in 2007. The cultured *K. pneumoniae* was from an infected cutaneous lesion of a 58-year-old man who had undergone multiple surgeries. The production of MBL was detected by phenotypic methods. Further testing showed *bla*IMP-1 and *bla*CTX-M-15 genes [20].

Later in 2008, Matar et al. reported the presence of the first *bla*OXA-48 *K. pneumoniae* in Lebanon in a 7-year-old female child with a history of recurrent urinary tract infection (UTI). The cultured *K. pneumoniae* was found to be resistant to imipenem and ertapenem [21].

In 2010, 2 major case reports on CRE were done at AUBMC. The first report studied 2 isolates; *K. pneumoniae* in a urine culture of a 47-year-old female patient with a community-acquired UTI and *E. coli* in a urine culture of a 49-year-old female patient with a community-acquired UTI. The *K. pneumoniae* isolate was found by PCR to be positive for the *bla-*CTX-M gene, a *bla*OXA-48-like gene, and the OmpF gene. On the other hand, the *E. coli* isolate was found to carry the *bla*TEM gene, a *bla*OXA-48-like gene, and the OmpF and OmpC genes [22]. This was an alarm that OXA-48-like positive Enterobacterales had become more widespread and even as community pathogens. The second report done that year was on three patients who were referred from Iraq. The blood culture of the first patient grew *E. coli* that was carbapenem resistant and harbored the OXA-48 gene. The blood culture of the second patient grew *K. pneumoniae* that coharbored OXA-48 and NDM-1. The final patient had *K. pneumoniae* in his urine culture which was NDM-1 positive. All three isolates also harbored OXA-1, TEM-1, and CTXM-15 with outer membrane porin mutations OmpF and OmpC [23]. This was the first report from Lebanon on CRE isolates imported from Iraq and showed that newer classes of CRE may start to circulate in the community.

In 2011, a retrospective study was conducted by Baroud et al. at AUBMC to study carbapenem resistance in ESBL-producing *K. pneumoniae* and *E.coli* isolates. Data showed that 1.07% of the ESBL-producing *E. coli* were nonsusceptible to ertapenem, with an MIC of 0.25 g/mL, and only 0.13% were resistant to all three carbapenems. PCR studies showed them to be positive for *bla*OXA-48 and its associated insertion sequence IS1999. On the other hand, 2.45% of the ESBL-producing *K. pneumoniae* isolates were ertapenem nonsusceptible with MIC ≥0.25 g/mL and only 0.13% were resistant to all 3 carbapenem and harbored *bla*OXA-48 and were positive for IS1999. Two of the cultured *K. pneumoniae* were from Iraqi patients and were found to harbor *bla*NDM-1 with one of them also being positive for OXA-48. An important finding was the fact that all the *E. coli* isolates harbored the ompF and/or ompC porin genes, whilst most of the *K. pneumoniae* isolates lacked these genes [24]. This study showed that the efflux pump activity plays a major role in carbapenem resistance in *E. coli* beside carbapenemase production.

The year 2012 witnessed the first broad surveillance of carbapenem-nonsusceptible Enterobacterales in isolates of hospitalized patients from different Lebanese governorates, with the majority being in Beirut and its suburbs. Hammoudi et al. started with 8717 Enterobacterales from which 102 were carbapenem nonsusceptible, but only 44 isolates were delivered to the central laboratory for further study. Thus, in 2012, there was around 1.2% CRE which indicates an increase in the percentage of its occurrence compared to the retrospective study in 2011 that was mentioned in the previous paragraph. From the 44 CRE sent for PCR, 31 (70.4%) harbored OXA-48 and 90.3% of them carried IS1999. One *K. pneumoniae* harbored OXA-48 with an acquired AmpC of the ACC type, which was first reported in Lebanon in this study [25, 26].

A retrospective study published later by Beyrouti et al. studied the endemic spread of OXA-48 as a cause of carbapenem resistance in Enterobacterales. A total of 2767 Enterobacterales isolates were recovered from clinical samples of hospitalized patients between 2008 and 2012, in addition to 183 fecal samples collected in 2012 from nonhospitalized school children who had not taken antibiotics in the previous 6 months. Data showed that resistance to ertapenem increased from 0.4% in 2008–10 to 1.6% in 2012 for the clinical isolates recovered from the hospitalized patients. A total of 24 nonredundant isolates with resistance to ertapenem were recovered between 2008 and 2012, and of these, 88% had the OXA-48 gene. In parallel to these results, 3 *E. coli* isolates from the fecal swabs (1.6%) had resistance to ertapenem with the OXA-48 gene [27].

Moghniyeh et al. did a retrospective study between 2009 and 2012 on multidrug-resistant Gram-negative bacteria causing bacteremia in febrile neutropenia adult cancer patients at Makassed hospital. 75 episodes of bacteremia were studied, and 45.3% of them were caused by Enterobacterales. Among the Enterobacterales isolates, imipenem resistance was detected in 8.8%, all being among *E. coli* isolates [28]. This study showed that patients who are undergoing chemotherapy are prone to more resistant bacteria including CRE especially due to the fact that they are subjected to antibiotics more often.

In the years 2011 and 2012, Hammoudi et al. monitored carbapenem resistance among Gram-negative strains at the Hôtel-Dieu de France hospital. A total of 466 isolates of *K. pneumoniae* and 139 isolates of *Serratia marcescens* were collected during the study. Data on *K. pneumoniae* showed a lower resistance to imipinem with 0.8% in 2011 and 0.9% in 2012. PCR testing further showed the cause to be the production of the *bla*OXA-48 gene and insertion sequence IS1999. Regarding *Serratia marcescens,* imipenem resistance rose from 3.6% in 2011 to 17.9% in 2012. PCR testing was only done on 4 isolates taken in 2012, and all were positive for the *bla*OXA-48 gene and insertion sequence IS1999. This was the first report to show such a high percentage of CRE (6.4% of all collected Enterobacterales), especially among the isolates of *Serratia marcescens* [29].

A retrospective study was done by Chamoun et al. on bacterial isolates from the bacteriology laboratories of 16 different tertiary care centers. A total of 44288 Enterobacterales were collected, being mostly *E. coli*. Data showed that *E. coli* had a lower rate of resistance to imipenem, with a mean resistance of 0.7% stable over the 3 year study period. *Klebsiella* spp showed 2% resistance to imipenem. This study lacked the genotypic study to further stratify those CRE, but it showed a shift to having more carbapenem resistance among *Klebsiella* spp which aligned with the worldwide data at that time [30].

Kissoyan et al. conducted a study between the years 2008 and 2014 at AUBMC to detect the prevalence of carbapenem resistance genes and their effect on the minimum inhibitory concentration required to inhibit the growth of 90% of the organisms (MIC90) on Enterobacterales. Nonduplicate ESBL-producing CRE *E. coli* (*n* = 76) and *K. pneumoniae* (*n* = 54) were collected and studied by PCR to detect genes responsible for the resistance. Data concerning the prevalence of CRE among *E. coli* and *K. pneumoniae* showed an increase between 2008 and 2014 from 0% in both to 1% and 4%, respectively. PCR experiments further clarified the culprit genes where 36% of all isolates had the *bla*OXA-48 gene (more in *E. coli*) and 18% of all isolates had the *bla*NDM-1 gene. Most of the *E. coli* isolates had at least one porin encoding gene, ompC or ompF (89% among *bla*NDM-1-positive isolates and 96% among *bla*OXA-48 isolates). These genes were found in lower proportions among *K. pneumoniae* isolates (27% among *bla*NDM-1-positive isolates and 65% among *bla*OXA-48 isolates) [31]. Another retrospective study was done at AUBMC between 2011 and 2014 and collected 40 CRE isolates. It was noted that 12.5% were community acquired and that 67.5% of the patients had received antibiotic therapy for >48 h in the previous 30 days [32].

The arrival of Syrian refugees post war was a factor in the sprea of CRE. In one report, the impact of this war on the susceptibility rates of Enterobacterales in Lebanon and Jordan between 2011 and 2015 was studied. A total of 702 Enterobacterales pathogens were collected from Lebanese isolates, with the majority being *E. coli* and *K. pneumoniae* from intra-abdominal infections (IAI) and UTI. The overall prevalence of carbapenem-nonsusceptible Enterobacterales was 1.8%. Further PCR studies showed that OXA-48 was found in 1.81% of the isolates in the year 2011, then dropped to 0% in the year 2013, and rose again to 1.7% in the year 2014, along with the appearance of OXA-244 in 1.7% of the isolates. The NDM gene was not found in any of the Lebanese isolates [33]. This study showed that the arrival of more Syrian refugees contributed to the rise of OXA-48 as shown in the studies mentioned in the previous paragraphs.

In the year 2016, the isolation of OXA-181-producing *E. coli* was reported. The sample was recovered from an 80-year-old male patient admitted to the ICU of AUBMC [34]. This gene was later discovered in other *E. coli* isolate that were collected in a retrospective study in AUBMC, also between 2012 and 2016. In the latter, Dagher et al. studied the molecular characterization of CRE and collected 27 *E. coli* isolates. All isolates were resistant to ertapenem, but only 22.2% had phenotypic resistance to meropenem. PCR analysis showed *bla*OXA-48 in 48.1% of the isolates and *bla*OXA-181 in 7.4% of the isolates. Around 44.4% of CRE had no carbapenemase encoding genes, and 75% of them showed multiple deletion events and truncations in ompC and ompF porin encoding genes [35]. This study showed a further slow spread of the newly discovered OXA-181 in Lebanon and verified the importance of changes in membrane permeability as a cause of CRE.

A new retrospective study between 2015 and 2016 compiled data from 13 Lebanese hospitals to examine the latest changes in antimicrobial resistance, including CRE. The data were compared to the surveillance report done by Chamoun et al. during the years 2011–2013. Data now showed a clear rise in CRE where 3% of *E. coli* were resistant to imipinem compared to 0.7% in the 2011–2013 surveillance and 4% of *K. pneumoniae* were resistant to carbapenem compared to 2% in the 2011–2013 report [36].

A 9-year retrospective study at Saint George Hospital in Beirut examined carbapenem-resistant organisms (CROs) from 2010 to 2018. A total of 2150 CROs were collected, with 15% being CRE throughout the study duration. It was noted that among all CROs, a general trend of increased imipenem resistance was observed starting in 2016. The percentage of CRE out of all CROs was 3% in 2010-2011 and increased to stabilize at an average of 8% until 2017. Then, in 2018, CRE quadrupled to 32% of CRO [14]. This study showed an epidemiologic shift among CROs from the dominance of nonlactose fermenters to the dominance of lactose fermenters.

In that same period of time, Mousally et al. studied the prevalence of antibiotic-resistant organisms in AUBMC between the years 2010 and 2018. 9447 Enterobacterales samples were cultured from various clinical specimens. Carbapenem-resistant *E. coli* (CREC) was 0% in 2010 and clearly rose through the years to reach an overall of 3.3% at the end of the study. This was similar to carbapenem-resistant *K. pneumoniae* (CRKP) that were also absent in 2010 and rose to reach an overall of 7.7% at the end of the study [37]. This study showed a further increase in the percentage of CRE, especially among *K. pneumoniae*.

During the period between 2015 and 2019, Moghnieh et al. compiled data from 1538 patients colonized and/or infected with third-generation cephalosporin-resistant (3GCR) Enterobacterales. 10% of the Enterobacterales acquired CRE throughout the study with an incidence density of 0.21 cases/1000 patient day at the beginning with an ascending trend reaching 1.89 cases/1000 patient day in 2019 [38]. This study showed again that the most common CRE pathogens were *Klebsiella* spp.

During the past two years, fewer studies about CRE were published, which could be attributed to the COVID-19 pandemic that largely affected the country [39]. A retrospective study was conducted between 2018 and 2019 in Zgharta Hospital in North Lebanon by Al-Bayssari et al. on 23 CRE isolates. Data showed that 56.5% of the isolates (the majority being *K. pneumoniae*) harbored the OXA-48 gene, 34.8% (all being *E. coli*) harbored the *bla*NDM-4 gene, 8.7% (all being *E. cloacae*) harbored the *bla*NDM-1 gene, and 8.7% (all being *K. pneumonia*e) harbored the *bla*NDM-6 gene along with OXA-48 [40]. This study was the first in Lebanon to identify the *bla*NDM-1 gene in *E. cloacae*. It further verified the increase in NDM genes among CRE isolates, which can be linked to the arrival of such genes from refugees in North Lebanon, as mentioned in studies above, and the fact that wastewater from that area also carried NDM genes.

Articles on CRE prevalence, mechanisms of resistance, and risk factors were also increasingly published in the neighboring counties. Taking the Arab League for instance, there were some studies that addressed the epidemiology of resistance among Enterobacterales in those countries. In this context, Moghnieh et al. studied CRE (among other resistant bacteria) in 14 Arab countries between 2008 and 2017. Throughout the whole study duration, the prevalence of CRE was found to be around 1% in Lebanon, KSA, and Qatar compared to around 2% in Algeria, Libya, Morocco, Mauritania, and Tunisia, 3% in Syria, 4% in Iraq, 22% in the Palestinian territories, 22.5% in Jordan, and the highest prevalence was 28% in Egypt. Despite having more *E. coli* samples collected, CRE was found more in *K. pneumoniae* isolates. Regarding PCR studies, data showed carbapenemase-producing genes to be mostly NDM-1- (46.5%) and OXA-48-like (32.5%). No KPCs were found in this data collection [41].

Another report in 2016 by Girmenia et al. reviewed the epidemiology of CRE in Mediterranean countries. Regarding KPC, the first outbreak in the Mediterranean region was reported in 2005 and continued to have an inter-regional diffusion later on. Egypt was the only other neighboring country to have some regional diffusion of KPC compared to an absence of this gene in isolates from Lebanon. Endemicity for KPC was found in Greece along the Mediterranean. As for MBL-producing isolates, Turkey and Greece had regional diffusion which was less profound than the inter-regional spread in Lebanon. Finally, regarding OXA-48, it was found that Turkey was endemic for this gene, while other neighboring countries had only some minor single outbreaks [42]. When compared to the data compiled so far from the Lebanese articles, this report shows that Lebanon is similar to the neighboring countries regarding MBLs but has a higher ratio of OXA-48-producing isolates.

A more recent study on the prevalence of CRE in the Eastern Mediterranean region was done by Sleiman et al. and was published in 2021. It was found that CRE prevalence in Iraq rose from around 12% in 2013 with OXA-48 being the most dominant to 66.1% as shown by a study done between 2014 and 2016 with the presence of *bla*OXA-48, *bla*NDM, *bla*VIM, and *bla*KPC genes. Similarly, in Iraq, the prevalence of CRE according to several studies done in 2018 was shown to be between 14% and 22%, with OXA-48 being the most common, with more expression of *bla*VIM genes lately. Regarding Palestine, CRE prevalence ranged from 13.3% to 35.5% in Gaza, with *bla*OXA-48 being scarce compared to the predominant *bla*SPM and *bla*IMP [43].

Globally speaking and from 55 countries, the SMART Global Surveillance Program from 2008 to 2014 collected a total of 103960 Enterobacterales isolates, of which 3.3% were nonsusceptible to ertapenem. 1.4% of all Enterobacterales was producers of carbapenemase and was mostly in *K. pneumoniae* isolates. PCR testing showed that 53.18% of CP-CRE were KPC positive, 20% were positive for OXA-48 like, 19.42% were positive for NDM, 6.16% had VIM, 2.67% had IMP, and only 0.2% were producers of GES [44]. Moreover, a bibliometric analysis from 2010 to 2020 was done by Zhong et al. including 1671 publications on CRE. Data showed CRE as a growing threat worldwide with an isolation rate of 10.32 per 100,000 hospital days. The most common isolated Enterobacterales were shown to be *K. pneumoniae* followed by *E. coli*. The predominant carbapenemase-producing genes found worldwide were KPC, NDM, and OXA types [45].

## 4. CRE Carriage in Healthcare Settings

As the prevalence of CRE was clearly rising, reports were done to pinpoint the possible causes of the spread. CRE carriage is considered a serious threat within healthcare settings where the spread mostly occurs. Patients colonized with CRE are reported to have an increased risk of developing an infection due to these pathogens [46–49]. Secondary bacteremia due to bloodstream invasion through a damaged mucosa was suggested as a route of infection in colonized patients. Carriers of carbapenemase-producing CRE in their digestive tract can spread carbapenemases, resulting in silent shedding [46]. In Lebanon, a high prevalence of MDR Enterobacterales carriage in nursing homes has been reported in two studies. The first was conducted in Beirut in two nursing homes and showed a high rate of MDR fecal carriage, reaching 80.7% of the participants. However, no carbapenem-resistant Enterobacterales were detected [50]. Dandachi et al. recruited 68 nursing home residents in Tripoli between December 2013 and April 2014 and did fecal swab cultures at regular intervals of 3-4 weeks. Data revealed a similar carriage rate of MDR Enterobacterales (76.5%). However, 1.7% (3 out of 178 MDR isolates) was carbapenem resistant with the OXA-48 gene [51]. Recent antibiotic use, urogenital pathologies, and diabetes mellitus were all linked to MDR fecal carriage [50, 51]. Regarding carriage in Lebanese hospitals, scarce data on CRE carriage were found. In the study conducted by Moghnieh et al. between 2015 and 2019, 41.3% of the patients who acquired CRE were only carriers. Five major risk factors for CRE acquisition were demonstrated: hematopoietic stem cell transplant recipient, previous cerebrovascular diseases history, chronic ulceration or wound, endoscopy within three months before hospitalization, acquisition of the CRE during hospitalization, and meropenem intake within 3 months of acquisition [38]. Another study conducted among cancer patients undergoing chemotherapy showed a 24.4% intestinal carriage rate of carbapenem-resistant bacteria, where *E. coli* was the most common isolated pathogen [52]. On the other hand, multiple studies were conducted worldwide to elucidate CRE carriage in hospitalized patients. Reports from Egypt [46, 53], Brazil, and Vietnam [54, 55] showed a high CRE carriage rate. Antibiotic abuse and length of hospital stay have been proposed as possible causes of this high carriage rate [46, 55]. Furthermore, CRE carriage was found to be prevalent in long-term acute care hospitals (LTACHs), postacute care hospitals (PACHs), and nursing homes [49]. Accordingly, an active surveillance program for the management of carriage through the implementation of a well-defined strategy relying on early identification of carriers, isolation of these patients, contact precautions, hand hygiene, and antimicrobial stewardship program are of utmost importance in order to control the spread of CRE. Thus, active surveillance testing through screening of patients who epidemiologically might not be linked to known CRE patients but meet certain prespecified criteria is mentioned as a supplemental measure in the Facility Guidance for the control of CRE in the updated CDC guidelines [8, 56, 57]. Screening should focus on high-risk patients, including not limited to patients admitted to hospitals from long-term acute facilities, other acute care hospitals, and those with a history of hospital admission within the last 12 months. In addition, severely immunosuppressed and critically ill patients in ICUs, hematological, transplants, major surgery, and infectious diseases units in which colonization is a major threat for invasive disease are preferred to be involved in an active screening [56]. In one study in a tertiary care center in China, active surveillance testing and program implementation over a period of three years resulted in a significant decrease in CRE infection [57]. However, data regarding the overall carriage rate of CRE and active surveillance program implementation in Lebanese hospitals are needed.

## 5. CRE Carriage in Community Settings

Even though the dissemination of CRE outside of healthcare settings has been rarely reported in developed countries, it has been documented in developing countries. Apparently, fecal-oral routes have been linked to the spread of CRE in communities, either by waterborne or foodborne transmission (53, 54). Contagion, described as the spread and dissemination of resistant bacteria and genes via contaminated potable water and poor sanitation, is a major driver of AMR, especially in low socioeconomic areas (58, 59). For instance, the contamination of surface and drinking water and the subsequent food chain by hospital wastewater is alarming and has been associated with a great potential to disseminate antibiotic resistance through colonization and infection by resistant strains (25, 26). To further address the issue, Daoud et al. studied wastewater from two hospitals with a total of 60 Enterobacterales collected from different pits in the years 2011 and 2012. In the hospital situated in Beirut, all *E. coli* and *Klebsiella oxytoca* isolates were ESBL producers, with *bla*OXA detected in the majority of the strains. It was only among *E. cloacae* that *bla*NDM-1 was positive in 25% of the isolates. In contrast, no carbapenemase genes were found in any of the isolates from the northern hospital [58]. The discovery of NDM-1 positive isolates may have a major impact on carbapenem resistance spread to the community.

Furthermore, the endemic status of bacterial carbapenemase producers in Lebanon can be elucidated and confirmed by estuary water contamination by *E. coli* and *K. pneumoniae* OXA-48 carbapenemase producers [59]. In addition, the fecal carriage of variable strains of OXA-48-producing *E. coli* was reported in one school in North Lebanon. Carriage was associated with a low socioeconomic level of carriers and highlighted the endemic state of CRE carbapenemase producers in the community, especially in low income neighborhoods [27].

Moreover, as mentioned previously, the Syrian conflict and the high rate of migration have been hypothesized as a possible source for the spread and outbreaks of antimicrobial-resistant infections [33]. Colonization and infections caused by bacteria with high rates of AMR have been reported among Syrian refugees. In Lebanon, multiple studies conducted in Syrian refugee camp environments showed the spread and emergence of AMR. The terrible humanitarian situation of the refugees with little hygiene and no reach to proper care, the overcrowded shared houses and shelters between families, and the absence of healthcare service for the large refugee population play a major role in the contagion role in AMR dissemination among the refugees community [60]. In particular, CRE carriage was detected in 20 out of 250 rectal swabs in a study conducted by Azour et al. in 2019 in two refugee camps in northern Lebanon. This study was the first to report NDM-1-producing *E. coli* carriage in healthy individuals [61]. Moreover, MDR *E. coli* isolation from wastewater in a screening conducted in the Al-Qaa refugee camp highlights the risk of AMR spread through the polluted environment, among the Syrian refugee population [60, 62].

## 6. CRE Prognosis and Mortality

Regarding prognosis, CRE infections are associated with higher mortality rates [63–65]. In Lebanon, a case series of CRE infections between 2011 and 2014 showed in hospital a mortality rate of 27.5%. However, several factors were potentially linked to mortality, such as the primary site of infection and antibiotic use 24 hours prior to CRE infection. Moreover, the number of complications was significantly higher in the mortality group and included sepsis, treatment failure, and respiratory failure [32]. Another recent study conducted between January 2015 and December 2019 showed a higher mortality rate, reaching 49.5% of CRE-infected patients. However, mortality rates were higher in respiratory infections and bacteremia in parallel with lower rates of clinical success. Moreover, the median length of hospital stay was higher in CRE patients in comparison to the carbapenem-sensitive Enterobacterales group. However, the absence of national standard guidelines and the unavailability of new recommended beta-lactam/beta-lactamase combinations for CRE-related infections played a key role in low treatment success [38] and explain this increase in mortality over time.

A meta-analysis conducted by Falagas et al. found similar mortality rate in CRE patients and it was between 26% and 44% [66]. Moreover, a more recent meta-analysis suggested inappropriate initial therapy as a possible cause for increased mortality in CRE patients [65]. Additional risk factors associated with mortality were identified in one case-controls study [67]. An initial, more severe comorbid conditions with a higher mortality risk in the presence of febrile neutropenia, septic shock and the absence of interventions for the infection focus treatment have been revealed as additional risk factors for mortality in patients with CRE [67].

## 7. Antimicrobial Misuse and the Role of Antimicrobial Stewardship

A worldwide large increase in antibiotic consumption has been reported in the last two decades, the highest being among the Middle East region. However, the greater antimicrobial consumption has favored the survival of resistant strains, as this consumption is considered as primary driver for antimicrobial resistance [68–70]. A meta-analysis done by Bell et al. found a close correlation between consumption and resistance. Consequently, increased antibiotics consumption is associated with greater resistance at community, national, and regional levels [70]. Exposure to antimicrobials prior to acquisition of CRE was demonstrated as a risk factor in multiple studies [46, 48, 71, 72]. In particular, a significant link was found between carbapenem consumption and CRE incidence in 2 studies conducted in China and Thailand. The greater carbapenem consumption over a period of five years was associated with a higher CRE rate [73, 74].

In Lebanon, antibiotic use prior to CRE infection in the majority of the patients was reported in one medical center in Beirut [32]. Furthermore, meropenem consumption was strongly correlated with CRE occurrence. Increased meropenem use was identified as an independent risk factor for CRE infection [60]. On the other hand, improper use and consumption in the Lebanese population is a major concern. In particular, the availability of antibiotics without a medical prescription, in addition to the overprescription by physicians, was shown to be frequent [75].

In order to minimize antibiotic misuse, the implementation of antimicrobial stewardship program (ASP) is needed in hospitals. The latter have a principle aim of optimizing the use of antibiotics for effective treatment of infections, decreasing adverse events caused by antibiotic misuse, and decreasing AMR emergence [76]. Consequently, previous ASP implementation resulted in a decrease in carbapenem use [77, 78]. In Lebanon, ASPs have been endorsed by the ministry of public health (MoPH) through its hospital's accreditation program and recommendations. However, the absence of clear national and in-hospital guidelines and the lack of regular education programs for medical staff, as well as infectious disease physicians shortage and minimal support from MoPH, were reported by Lebanese physicians as barriers for ASPs in Lebanon [79]. Moreover, the control of outpatients' antibiotics prescription should be addressed since most of outpatients' antibiotic prescriptions are broad spectrum and can be accessed without a medical prescription [80, 81]. Moreover, prescriptions without a clear indication, inadequate dosage, and incorrect duration of therapy were frequently found in a recent cohort study in the Lebanese community [81].

In conclusion, the situation of CRE among the Lebanese community and healthcare settings is alarming. There is an increase in CRE infections and carriage as well as the emergence of new classes of carbapenemases other than the predominant class D OXA-48 carbapenemases. Moreover, the Syrian conflict and refugee crisis, water contamination, and widespread antimicrobial misuse are among the major causes of the dissemination of antimicrobial resistance, including CRE, among the Lebanese community. More strict infection control measures in healthcare settings, in addition to accurate ASP implementation, are urgently needed.

## Figures and Tables

**Table 1:** A summary of all detected carbapenemase types in Lebanon since the first reported carbapenemase in 2007. IMP: imipenemase metallo-*β*-lactamase; NDM: New Delhi metallo-*β*-lactamase; OXA: oxacillin carbapenemase/oxacillinase.

Years	Studies	Types of detected carbapenemase
2007	Daoud et al.	IMP
2008	Matar et al.	OXA-48
2010	El-Herte et al.	OXA-48 and NDM-1
2011	Baroud et al.	OXA-48 and NDM-1
2011-2012	Hammoudi et al.	OXA-48
2012	Hammoudi et al.	OXA-48
2012	Beyrouti et al.	OXA-48
2008–2014	Kissoyan et al.	OXA-48 and NDM-1
2011–2015	Hajj et al.	OXA-48 and OXA-244
2012–2016	Dagher et al.	OXA-48 and OXA-181
2018-2019	Bayssari et al.	OXA-48, NDM-1 and NDM-4
